# The impact of paternal alcohol, tobacco, caffeine use and physical activity on offspring mental health: a systematic review and meta-analysis

**DOI:** 10.1186/s12978-021-01266-w

**Published:** 2021-10-26

**Authors:** Kayleigh E. Easey, Gemma C. Sharp

**Affiliations:** 1 MRC Integrative Epidemiology Unit at the University of Bristol, Bristol, UK; 2 Department of Population Health Sciences, Bristol Medical School, University of Bristol, Bristol, UK

**Keywords:** Systematic review, Mental health, Tobacco, Alcohol, Caffeine, Physical activity, Fathers, Paternal, Pregnancy, Child health, Meta-analysis

## Abstract

**Background:**

There is some evidence that paternal health behaviours during and around pregnancy could be associated with offspring health outcomes. However, the impact that paternal health behaviours during pregnancy can have on offspring mental health is understudied and remains unclear.

**Methods:**

We conducted a systematic review and meta-analysis of articles in PubMed describing studies of potentially modifiable paternal health behaviours (tobacco smoking, alcohol consumption, caffeine consumption and physical activity) in the prenatal period in relation to offspring mental health. GRADE was used to measure risk of bias.

**Results:**

Eight studies were included and categorized by paternal health behaviour and offspring mental health outcome investigated. The narrative synthesis provided evidence of association between paternal health behaviours around pregnancy and offspring mental health problems, with the strongest evidence shown for tobacco use. Grouped by analysis type, two separate meta-analyses showed evidence of paternal smoking during pregnancy being associated with greater odds of ADHD in offspring (OR 1.42, 95% CI 1.02–1.99; HR 1.28, 95% CI 1.19–1.39).

**Conclusions:**

The small number of studies that have investigated paternal prenatal effects on offspring mental health, and the limited sample sizes of those studies, makes it challenging to draw firm conclusions. Although existing studies suggest that paternal tobacco smoking and alcohol consumption in the prenatal period are associated with poorer offspring mental health, (particularly hyperactivity/ADHD), further investigation of potential paternal effects is required, using methods that allow stronger inference to determine whether associations are causal.

**Supplementary Information:**

The online version contains supplementary material available at 10.1186/s12978-021-01266-w.

## Plain language summary

More research has focused on the impact mothers’ behaviours (such as smoking or alcohol use) during and around pregnancy may have on their children’s health, with less research investigating the role paternal health behaviours may play.

This review captured what research was currently available that investigated the impact of paternal alcohol, tobacco, caffeine use, and physical activity during pregnancy on children’s mental health.

We showed that this area is currently under researched, finding only eight studies. However, of the research that was already published we found evidence of paternal health behaviours having an impact on children’s mental health. The strongest evidence was shown for paternal smoking during pregnancy having a negative impact on children’s hyperactivity/ADHD. No studies measured paternal caffeine use or physical activity around pregnancy. This review highlights the lack of research that has investigated the association between paternal modifiable health behaviours around pregnancy and offspring mental health. Despite including four different types of paternal health behaviours and a broad definition of offspring mental health across any age, only eight studies were shown. This review suggests further research within this area is needed which may influence health warnings to potential fathers to be both before conception and during pregnancy.

**Supplementary Information:**

The online version contains supplementary material available at 10.1186/s12978-021-01266-w.

## Introduction

The Developmental Origins of Health and Disease (DOHaD) literature has overwhelmingly focused on how maternal health behaviours during pregnancy may causally impact offspring health, including offspring mental health [1–3]. Far less research has assessed the potential causal effect of paternal exposures [4, 5], although there is some evidence that paternal health behaviours during and around pregnancy could be associated with offspring health outcomes [6, 7].

Paternal traits and behaviours could influence offspring health through genetic inheritance and environmental influences, both directly and indirectly. Direct paternal effects can occur due to epigenetic changes within spermatozoa caused by environmental factors [8]. Paternal diet or smoking can cause damage to DNA and de novo mutations within the male germline which could then directly influence offspring phenotypes [7], for example high-fat diets have been shown to reduce sperm counts and mobility [9]. Germline transmission of epigenetic modifications could also directly influence offspring health. However, there are also indirect pathways whereby paternal behaviours may influence the maternal environment, behaviour and physiology, which could then influence offspring outcomes via intra-uterine mechanisms. Direct prenatal paternal effects can only occur at pre-conception, whereas indirect effects can occur during pregnancy [7]. Second-hand smoke exposure from paternal tobacco use during pregnancy is able and likely to impact the health of both mother and child [10, 11]. However, paternal use of alcohol, caffeine, or physical activity is unable to influence intrauterine development, except via a small indirect effect on maternal behaviour, such as by encouraging/discouraging exercise or not supporting mothers to abstain from alcohol or caffeine during pregnancy. For example, paternal support in reducing tobacco use during pregnancy has been shown to be a key component in mothers cessation also [12], particularly when partners support has been high [13]. There is need to study these other paternal exposures during pregnancy to help to contextualise the maternal effect and as a negative control to explore causality. Negative control analyses are a method used to explore if associations are due to confounding or are likely to be causal [14], and are often the main reason why paternal exposures during pregnancy have been included in previous research [15, 16]. A recent review distinguished between the impact of direct paternal biological and environmental effects, again highlighting the way paternal behaviour can impact offspring health [17]. This review was broad, focusing on outcomes of male fertility, early pregnancy complications as well as fetal and postnatal outcomes. Although the previous review did find evidence of paternal factors having a detrimental effect on offspring neurodevelopmental disorders, this was shown only in relation to paternal exposures such as medication use or diagnoses of poor health. It was therefore not designed to specifically capture either mental health outcomes in offspring, or modifiable paternal health behaviours such as alcohol or tobacco use during pregnancy.

Another review of the literature on paternal effects on child obesity and type 2 diabetes found some support for paternal influence on these outcomes, but also highlighted the paucity of high-quality research being conducted within this area [7]. Findings from a review from 2018 found evidence of associations of paternal age and paternal smoking with preterm birth, low birthweight and several congenital anomalies [18]. The strongest associations shown were between paternal age and autism/autism spectrum disorders (pooled adjusted odds ratio per 5-year age increase: 1.25; 95% CI: 1.20–1.30) and schizophrenia (OR: 1.31; 95% CI: 1.23–1.38). However, it is unclear whether these and other psychiatric and mental health conditions are associated with potentially modifiable paternal health behaviours, like tobacco smoking, alcohol consumption, caffeine consumption and physical activity.

In this systematic review, we summarise the literature on associations of these paternal health behaviours in the prenatal period with offspring mental health outcomes. This provides insight into whether (and which) paternal prenatal health behaviours are likely to causally influence offspring mental health.

## Methods

This review was conducted according to PRISMA (Preferred Reporting Items for Systematic Reviews and Meta-analyses) guidelines [19], and preregistered on the Open Science Framework (*osf.io/adnbu*). We conducted a systematic search of PubMed to identify publications up until 01.03.2021.

### Search strategy

The search strategy included key words related to “paternal” (Paternal OR father* OR dad* OR partner* OR intergenerational) and “mental health” (‘mental health’ OR depress* OR anxiety OR mood OR internali?ing OR externali?ing OR conduct OR ADHD OR attention OR hyperactiv* OR ‘emotional problems’) and “offspring” (child* OR offspring OR son* OR daughter*) and “health behaviours” (smok* OR tobacco OR cigar* OR alcohol* OR caffeine OR coffee OR exercise* OR ‘physical activity’). We specified that these words should appear in the title or abstract of publications, see Additional file 1: Methods for the exact search terms used.

Eligibility was defined using the PICOT framework:

Population: Fathers or mothers partners of study children, non-animal studies.

Intervention: Paternal alcohol, tobacco, caffeine use or physical activity.

Comparison: Paternal non-exposure of corresponding health behaviour.

Outcome: Any measurement of offspring mental health.

Timeframe: Pre-conception (up to six months pre-pregnancy) and during pregnancy.

### Eligibility criteria

Studies were excluded if they were review articles with no original data or had not studied the exposures or outcomes of interest. Any source of mental health measure was included (e.g., self-report or parental report) and offspring outcomes could be measured at any age. Studies were only included if paternal exposures were measured for pre-conception or during pregnancy.

### Study selection and data extraction

One reviewer (KEE) assessed studies for inclusion/exclusion based on the title and abstract and full text. Another reviewer (GCS) assessed a random 10% of studies for inclusion/exclusion using the same criteria. For included studies, one reviewer (KEE) extracted data on study location, design, paternal exposure, exposure timepoint, mental health outcomes, offspring age, included covariates, sample size, whether both parents were studied, species studied, statistical methods and results. Where the above data was missing from manuscripts, we contacted authors of the original studies for further information. If included studies measured multiple mental health outcomes, the data were extracted separately for each outcome. We present the most covariate-adjusted results from the original study that were available. A measure of strength of evidence was included for each study using the Grading of Recommendations Assessment, Development and Evaluation (GRADE) methodology [20]. The quality of evidence for each study was assessed on risk of bias (limitations due to study design), consistency, directness (appropriate sample used), precision (95% CI), and publication bias. Resulting in assessment of very low, low, moderate, or high quality of evidence.

### Meta-analysis

Fixed effects meta-analysis was conducted where appropriate (i.e., where exposures and outcomes were measured and defined similarly, and similar statistical tests were used to derive study estimates). Analyses were conducted using the *metan* command within Stata version 15. Between study heterogeneity was assessed using *I*^2^.

## Results

### Narrative synthesis

**Fig. 1:**
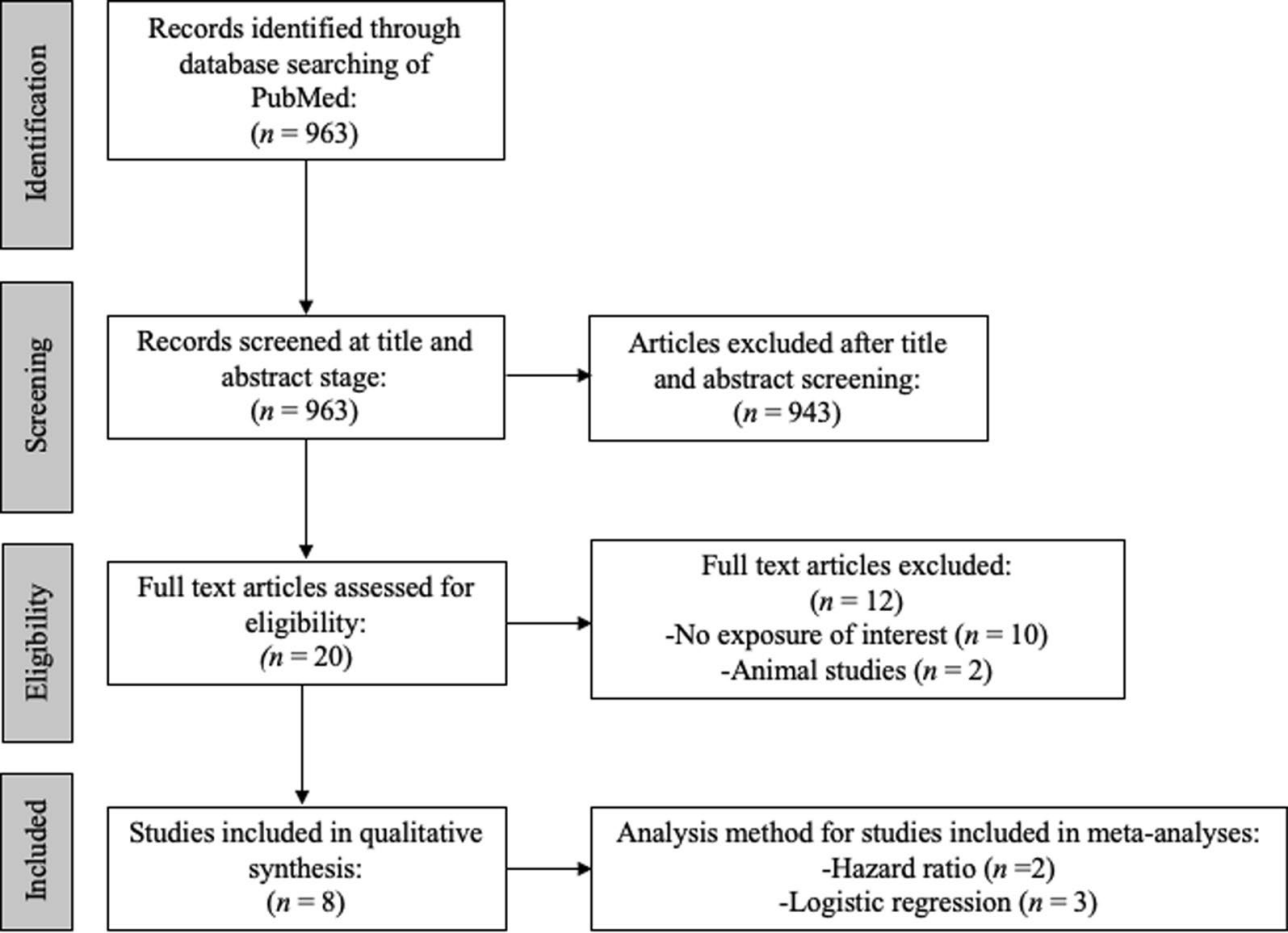
Flowchart of search strategy

The initial search identified 963 articles, of which 943 were excluded based on title and abstract. Of the 20 that underwent full text review, 12 did not meet the inclusion criteria (did not measure paternal exposures in pregnancy/pre-conception), see Fig 1. Offspring age varied from four to 24 years. Two studies were conducted in the USA, two in the UK, and one in each of Norway, Denmark, the Netherlands and Germany.

There was considerable heterogeneity between studies regarding measures of paternal exposure and offspring mental health outcome, length of follow-up, statistical methods used, and confounders adjusted for. Of the eight included studies, seven included both maternal and paternal exposures using paternal exposures as a negative control analyses to explore causality [15, 21–26]. The remaining study measured paternal exposure only [27]. All included studies were observational and used a human sample.

### Assessment tools used

Of the eight included studies, seven studied paternal tobacco use as an exposure, and one studied alcohol use. There were no studies measuring paternal caffeine use or physical activity. Seven studied paternal exposures during pregnancy and one just before pregnancy initiation. The studies considered seven different measures of offspring mental health (often more than one outcome was considered). ADHD and/or hyperactivity subscales were investigated by seven studies. Depression, Oppositional Defiant Disorder (ODD), conduct disorder, emotional problems and a total problem score (combining multiple problems) were considered by one study each. See Table 1 for an overview of included full text studies.

**Table 1:** Studies included after full text screening

Study	Data source	Location	Exposure	Exposure timepoint	Outcome	Offspring age	Sample size	Covariates	Results	Strength of evidence*
Gustavson et al. (2017) [21]	MoBa	Norway	Smoking	Pregnancy	ADHD	5 years	104,846	Maternal and paternal age, maternal and paternal education, maternal and paternal ADHD symptoms, maternal (pre-pregnancy) and paternal BMI, maternal alcohol consumption during pregnancy, parity, child’s birth year, geographical region	OR: 1.28, CI: 1.16–1.42	Moderate
Biederman et al. (2020) [27]	NA	USA	Smoking	Pre-conception	ADHD	6–18 years	226	Paternal ADHD	OR: 1.50, χ2: 1.58, p = .21	Low—due to risk of bias
Langley et al. (2012) [22]	ALSPAC	UK	Smoking	Pregnancy (18 weeks gestation)	ADHD	7.6 years	8324	Child's sex, ethnicity, multiple births (twins), maternal alcohol use during pregnancy, social class	Number of ADHD symptoms. Paternal in separate model: β: 0.17 (CI 0.11, 0.23), p < 0.001. Both parents included in same model: β: 0.14 (CI 0.07, 0.20), Paternal smoking where mother is a non-smoker: β: 0.12 (0.04, 0.20), p < 0.001; fathers: β = 0.06, 95% CI: 0.03, 0.09). Diagnoses of ADHD: OR:1.43 (CI 0.98, 2.07)	Moderate
Zhu et al. (2014) [23]	DNBC	Denmark	Smoking	Pregnancy (16 weeks gestation)	ADHD	8–14 years	84,803	Maternal age, parity, alcohol intake during pregnancy, parental socioeconomic status, parental psychopathology, child’s sex	HR: 1.29 (1.14 to 1.47)	Moderate
Altink et al. (2009) [24]	International Multi-centre ADHD Gene project (IMAGE) and controls	The Netherlands	Smoking	Pregnancy	Attention	12.5 years	79	Age, sex, IQ, birth weight, oppositional symptoms of the child, anxious-shy symptoms, total maternal or paternal ADHD symptoms, maternal age, socio economic status	Main effect: F(1,86.8) = 7.62, P = 0.007	Moderate
Nomura et al. (2010) [25]	Longitudinal study of children at risk for ADHD	USA	Smoking	Pregnancy	ADHD, ODD	4.3 years	209	Age, sex, SES, birth weight, race of the child, self-report maternal and paternal ADHD symptoms, maternal alcohol use during pregnancy	ADHD: OR: 0.31 (0.06–1.92), p = 0.21. ODD and ADHD COMORBID: OR: 0.85 (0.13–5.55), p = 0.86	Low – due to imprecision
Tiesler et al. (2011) [26]	LISAplus	Germany	Smoking	Pregnancy	Total problems, emotional difficulties, conduct problems, hyperactivity, peer problems	10 years	1654	Sex, study centre, parental education, mother's age at birth, time in front of screen and having single mother/father	Total difficulties: OR: 1.21 (0.45–3.27). Emotional problems: OR: 1.54 (0.73–3.27). Conduct probs: OR: 0.73 (0.30–1.77). Hyperactivity/inattention: OR: 2.03 (0.86–4.81). Peer probs: OR: 1.22 (0.51–2.91)	Low—due to imprecision
Easey et al. (2020) [15]	ALSPAC	UK	Alcohol	18 and 32 weeks gestation	Depression	18 and 24 years	2566	Socioeconomic position, income, homeownership, marital status, maternal education, gender, parity, maternal tobacco use during 1 to 3 months of pregnancy, maternal illicit drug use during 1 to 3 months of pregnancy, maternal depression 18 weeks gestation, how often partner consumed alcohol at 18 weeks gestation	Age 18: Alcohol frequency: OR 0.87, 95% CI 0.74 to 1.01, p = Pattern (binge): 0.95 (0.87–1.03). P = 0.194 Age 24: Alcohol Frequency: OR .02 (0.89–1.16) 0.790. Patten (binge) OR: 0.99 (0.91–1.07), p: 0.771	Moderate

*HR* Hazard ratio, *OR* Odds ratio

*Using Grading of Recommendations Assessment, Development and Evaluation (GRADE) methodology

### Included study characteristics

Of the eight included studies, four (50%) found evidence of paternal health behaviours around pregnancy being associated with offspring mental health. The four (50%) remaining studies reported no clear evidence of association; three of these studies measured paternal smoking and one of paternal alcohol use. All the included studies which reported associations between paternal substance use and offspring mental health problems; all of these studied paternal smoking.

### Quality of evidence

Five of the eight studies showed moderate quality of evidence, and three showed low precision within their results (see Table 1). A low quality of evidence score was given mainly due to low sample size and limitations in study design. Specifically, all studies were observational and only three studies used mutual adjustment of maternal behaviour to account for assortative mating.

### Meta-analysis

**Fig. 2:**
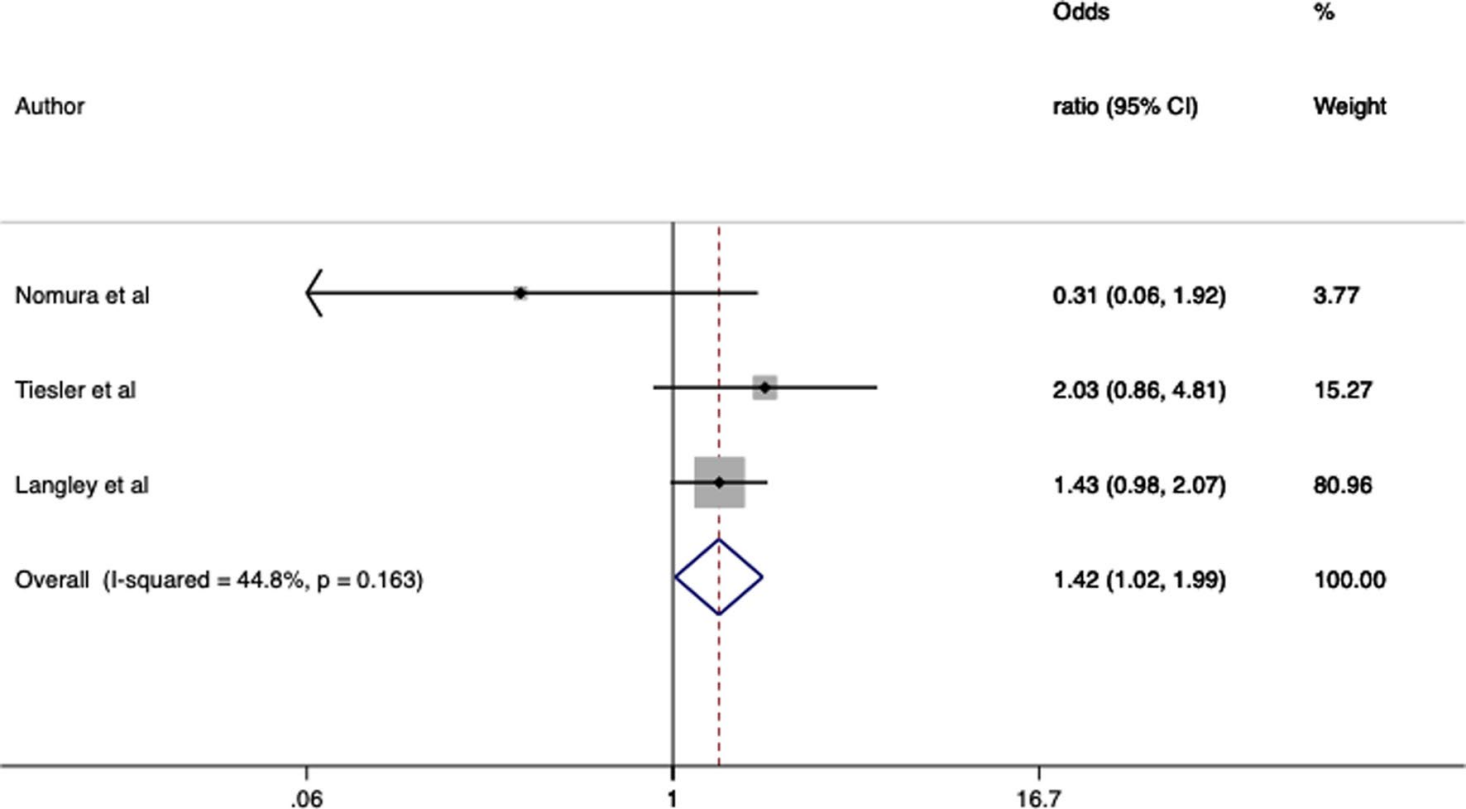
Forest plot of associations between paternal smoking in pregnancy and offspring ADHD of studies reporting odds ratios

**Fig. 3:**
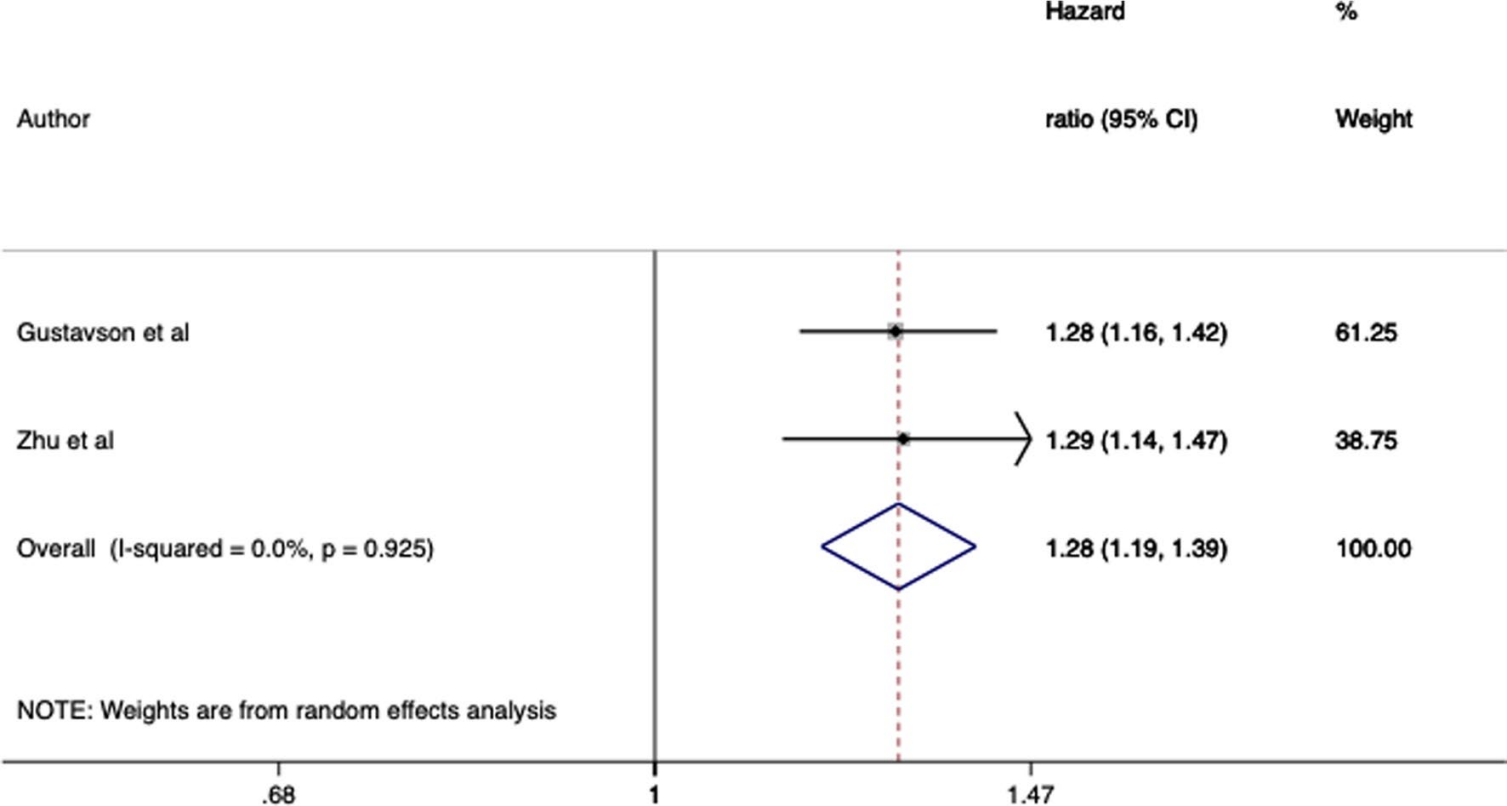
Forest plot of associations between paternal smoking in pregnancy and offspring ADHD of studies reporting hazard ratios

There was enough comparable data from studies of paternal smoking during pregnancy and offspring ADHD to conduct two meta-analyses: one for the 3 studies that reported odds ratios from logistic regression, and one for the 2 studies that reported hazard ratios. One additional study that met our criteria was not included, as following our request the corresponding author declined to provide additional statistical information required for the meta-analysis. Both meta-analyses found evidence that paternal smoking during pregnancy was associated with increased ADHD in offspring (logistic regression meta-analysis: OR 1.42, 95% CI 1.02–1.99, *I*^2^ = 45%; hazard models meta-analysis: HR 1.28, 95% CI 1.19–1.39, *I*^2^ = 0%), see Fig. 2 and 3.

## Discussion

The aim of this systematic review was to investigate the association between paternal health behaviours (alcohol, tobacco, caffeine, physical activity) around pregnancy and offspring mental health, and to quantify the degree to which published research has considered mental health in relation to these paternal prenatal health behaviours. Within our narrative review, we found limited research investigating the influence of paternal exposures on offspring mental health. Half of the studies included reported increased amounts of tobacco use during pregnancy to be associated with detrimental offspring mental health outcomes, the other half reported no clear association. This review highlights the paucity of research that has investigated the association between paternal modifiable health behaviours around pregnancy and offspring mental health. Despite including four health behaviours as potential paternal exposures and using a broad definition of offspring mental health across any age, only eight studies were eligible for inclusion. By contrast, two reviews of maternal alcohol use and offspring mental health identified 26 [1] and 8 studies [30]. The lack of published research studying paternal effects and offspring mental health makes it challenging to draw conclusions from the available data. Most studies focused on paternal smoking above other paternal behaviours, and hyperactivity/ADHD was by far the most studied offspring outcome. Only one study did not investigate tobacco use, and only one did not study hyperactivity/ADHD. This is unsurprising given that of the exposures we measured, paternal smoking is the only behaviour that can have a direct intrauterine effect (via passive smoke) [7]. This trend for research to focus on behavioural problems may not actually be unique to paternal exposures. A previous review of maternal alcohol use during pregnancy and offspring mental health has shown similar trends, with the majority of studies shown to measure offspring behavioural problems of conduct disorder, with less focusing on internalising problems [1]. Of the studies included in the current review, half found paternal behaviours of smoking use to be associated with negative offspring mental health outcomes. Paternal smoking during pregnancy was shown within the narrative synthesis and meta-analysis to be associated with increased rates off ADHD in offspring. There were no studies which evaluated paternal caffeine use or physical activity in or around pregnancy, highlighting an under researched area in which future research is needed, to investigate if there are any (albeit potentially small) indirect effects of caffeine use/physical activity on offspring mental health. Although animal studies were excluded from the current review, we did identify two animal studies during our search which investigated these paternal exposures on offspring mental health. One study indicated that increased paternal alcohol use was associated with offspring hyperactivity [28]. The other showed increased physical activity in the pre-conception period to be protective for offspring mental health by reducing anxiety [29]. Own exercise has been associated with a reduction in mental health problems in humans [31, 32], therefore this potential intergenerational effect (of either paternal or maternal physical activity) warrants further investigation. Although animal studies can enable greater control and precision in measuring and manipulating the exposure, findings cannot be directly related to human populations, where exposures, outcomes, physiology, and social contexts are very different. Therefore, further research in human samples is needed to confirm findings from the animal studies we identified.

In general, our review suggests that paternal health behaviours around pregnancy may have an impact on offspring mental health, but that this area is very under researched. There are limitations within the included studies which can make causal interpretation challenging. In particular, the number of studies within each meta-analysis was small. This could mean that the results from each meta-analysis are more representative of a selected sample only. Included studies varied wildly in terms of sample sizes, offspring ages, exposures and outcome measures. Furthermore, although we have presented results from fully adjusted models from each original study, there were inconsistencies in the confounders included in statistical models, which could contribute to inter-study heterogeneity in estimates. It is therefore unclear if residual confounding may be accounting for some results. Additionally, across included studies only three studies adjusted for the measured paternal health behaviour in the mother (e.g., studies of paternal smoking adjusting for maternal smoking). When studying any parental effect, mutually adjusted models help to account for exposure related assortative mating. Failing to make mutual adjustments for maternal and paternal exposures can result in bias from assortative mating [33, 34]. By not adjusting for maternal pregnancy exposures, it cannot be certain that any associations with paternal exposures are not due to maternal contribution. Of the three studies that accounted for assortative mating through mutual adjustment, two showed paternal smoking to be associated with offspring ADHD, and the remaining study which measured paternal alcohol use and offspring depression showed no clear association. Future research using mutually adjusted models is needed to ascertain if the difference in associations shown between these behaviours are due to behaviour type and the inability for paternal alcohol use having a direct intrauterine affect, compared to passive smoke exposure as previously discussed.

The limited number of studies shown from this systematic review, makes interpretation and generalizability of the findings challenging. Further good quality research is required to understand the potential causal impact of paternal health behaviours around pregnancy. Within the included studies we found the quality of evidence to vary between studies. Six studies showed moderate quality of evidence, however four of the studies showed low precision within their results. This variation in strength of evidence between studies was mainly due to limited sample sizes meaning results were imprecise. A low quality of evidence was also shown within studies due to lack of mutual adjustment of maternal behaviour, as previously discussed. Studies with a moderate quality of evidence, included appropriate adjustment for potential confounding as well as high precision. Future research including larger samples of paternal exposures which also adjusted for maternal behaviour would be beneficial to increase future certainty in reported results.

Aside from the low number of studies found within this area, there are other limitations which must be considered when interpreting these results. First, many of the included studies are observational and whilst they can identify associations, they do not alone provide evidence of causality. Causal inference is challenging due to the well-described problems of confounding and residual confounding in observational research [14]. Second, which timepoint paternal health behaviours were measured within pregnancy/pre-conception was varied, with some studies only stating measures were obtained *during* pregnancy. This means we are unable to conclude if paternal exposures during any particular stage of pregnancy or pre-conception has a greater impact on offspring mental health. Third, the systematic review search was conducted within PubMed only. Previous research has shown that the use of a single database does not bias study inclusion [35, 36], and attempts to define a set standard for the amount of databases to used found no firm conclusion [37]. However, we acknowledge that using a single database for our search may have resulted in relevant studies not necessarily being found. Fourth, all studies included within this review were conducted using a western sample from developed nations. This may limit the generalizability of the findings to low- and middle-income countries where substance use behaviours and confounding structures may differ. Further research within this area would be welcomed using more globally representative samples. Lastly, in an attempt to assure the quality of included studies, articles were only included in this review if they were already published in peer-reviewed journals. However, the peer-reviewed scientific literature can suffer from publication bias whereby studies reporting null results are less likely to be published.

In summary, we have identified eight eligible studies of paternal health behaviours in relation to offspring mental health, four of which found some statistical evidence of association. Mirroring the focus in the literature on maternal effects on offspring mental health, most studies were of tobacco use during pregnancy, and most considered offspring ADHD/hyperactivity over internalizing behaviours. It is notable that we identified only a small number of studies that considered paternal exposures. This further highlights the imbalance of DOHaD research towards studies of maternal pregnancy exposures, which has been illustrated by other recent studies [4, 5]. This review adds to research demonstrating the influence of paternal health and lifestyle on offspring health [6, 7, 17], and specifically the detrimental impact paternal behaviours around pregnancy may have on offspring mental health.

Given the potential for studies of paternal exposures to reveal important causal paternal effects, and to help contextualise the large body of maternal effects literature, further research is needed to investigate the causal impact of paternal health behaviours on mental health. Lack of evidence on the causal impact of paternal prenatal behaviours on offspring health may mean we are missing out on a potential pathway to reduce harm. Further research within this area could therefore influence paternal health warnings and advice for fathers during pregnancy and potential fathers to be during pre-conception.

## Supplementary Information

**Additional file 1.** Additional methods.

## Data Availability

Not applicable.

## Abbreviations

DOHaD: The Developmental Origins of Health and Disease
ADHD: Attention Deficit Hyperactivity Disorder
